# The Exponential Shapeshifting Response of N-Vinylcaprolactam Hydrogel Bilayers Due to Temperature Change for Potential Minimally Invasive Surgery

**DOI:** 10.3390/jfb15090242

**Published:** 2024-08-24

**Authors:** Billy Shu Hieng Tie, Mark Daly, Shuo Zhuo, Elaine Halligan, Gavin Keane, Joseph Geever, Luke Geever

**Affiliations:** 1 Polymer, Recycling, Industrial, Sustainability and Manufacturing (PRISM) Centre, Technological University of the Shannon, Midlands Midwest, N37 HD68 Athlone, Ireland; a00238048@student.ait.ie (S.Z.); a00218425@student.tus.ie (E.H.); 2 Faculty of Engineering & Informatics, Technological University of the Shannon, Midlands Midwest, N37 HD68 Athlone, Ireland; mark.daly@tus.ie (M.D.); joseph.geever@tus.ie (J.G.); 3 Centre for Industrial Service & Design, Technological University of the Shannon, Midlands Midwest, N37 HD68 Athlone, Ireland; gavin.keane@tus.ie; 4 Applied Polymer Technologies Gateway, Technological University of the Shannon, Midlands Midwest, N37 HD68 Athlone, Ireland

**Keywords:** temperature-responsive, hydrogels, smart materials, smart actuator, photopolymerisation, soft materials, hydrophilic, lower critical solution temperature

## Abstract

Poly (N-vinylcaprolactam) (PNVCL) and poly (N-isopropylacrylamide) (PNIPAm) are two popular negatively temperature-responsive hydrogels, due to their biocompatibility, softness, hydrophilicity, superabsorbency, viscoelasticity, and near-physiological lower critical solution temperature (LCST). These characteristics make them ideal for biomedical applications. When combined with other materials, hydrogel expansion induces the morphing of the assembly due to internal stress differences. Our recent developments in NVCL hydrogel, enhanced by nanoclay incorporation, have driven us to the creation of a bilayer structure to study its shapeshifting response across various temperatures. This study focused on the bending behaviour of bilayer samples composed of an active hydrogel layer and a passive non-swellable layer. Using photopolymerisation, circular discs and rectangular bilayer samples of varying sizes were fabricated. Homogeneous circular samples demonstrated that hydrogel density increased proportionally with temperature, with the swelling ratio exhibiting two distinct rates of change below and above its LCST. In bilayer samples, the volume of the passive layer influenced bending, and its optimal volume was identified. The investigation revealed that geometry affected the overall bending effect due to changes in the passive layer stiffness. Lastly, a temperature-responsive gripper capable of picking up objects several times its own weight was demonstrated, highlighting the potential of NVCL hydrogels as bioactuators for minimally invasive surgery.

## 1. Introduction

Hydrogels are a group of materials that swell in water due to the absorption of water into their polymer network [1]. When hydrogels’ swelling behaviours are affected by external stimuli such as pH, temperature, light, electricity, etc., they are identified as stimuli-responsive or smart hydrogels [2,3,4]. Smart hydrogels, along with shape memory materials [5,6], auxetic structures [7], liquid crystalline elastomers (LCEs) [8], and ferrofluids [9], are considered stimuli-responsive or smart shapeshifting materials. These materials react to external stimuli and undergo a series of changes, eventually leading to a shape change. In brief, when compared to hydrogels’ shapeshifting mechanism of expansion through water absorption, shape memory materials exhibit the shape memory effect that allows them to be transformed into their pre-programmed shape after being stimulated [10]; auxetic structures possess a negative Poisson’s ratio, enabling them to be expanding transversely under tension [11]; LCEs have the characteristics of both elastomers and liquid crystals, showing entropy elasticity and the ability to self-organise [12]; and ferrofluids demonstrate a rapid response to magnetic reactions by modifying their droplets’ shape depending on the magnetic field strength they are exposed to [13]. There is a growing interest in research on smart shapeshifting materials due to their potential to be used in various practices, including biomedical applications [14], soft robotics [15], sensors [12], soft actuators [16], etc.

While other smart shapeshifting materials can generally shapeshift independently, hydrogels usually need to be combined with other materials to achieve sophisticated shapeshifting beyond mere expansion. To achieve bending or buckling, hydrogel programming strategies that are typically used include creating multilayer, material gradients; localised activation; material tessellation; in-plane material gradients; and mechanically induced buckling [17]. The most common programming strategy for creating shapeshifting hydrogels involves multilayer structures, utilising either homogeneous or non-homogeneous materials to form isotropic or anisotropic layers with the hydrogels. Multilayer hydrogels can execute a variety of shapeshifting mechanisms, such as rolling, helixing, bending, folding, twisting, etc., to meet diverse application needs. Examples of multilayer hydrogels are self-folding grippers [15], self-folding origami [18], microrobots [19], and delivery models for cartilage regeneration [20]. Considering the other programming strategies, Huang et al. demonstrated the fabrication of printed hydrogel sheets using varying durations of UV light exposure, which resulted in the formation of 3D structures due to the generation of localised stress caused by different swelling degrees [21], and Kim et al. created thermally responsive rolling thin gel strips by applying two different dosages of UV irradiation with the aid of photomasks [22]. In fact, to achieve complex shapeshifting rather than simple expansion, materials that are combined with hydrogels must exhibit different responses when placed in water, e.g., a bilayer structure consisting of a non-swellable passive layer and a swellable active layer. Hence, the difference in swelling/shrinkage between both layers can result in the shapeshifting ability, such as the bending of the bilayer structure [17].

When programming smart shapeshifting materials, usually, materials’ behaviours before and after stimulation must be studied first. This ensures that the materials achieve the intended changes in shape, properties, or functionality. Hence, Momeni et al. stated that in 4D printing, the fundamental components are a 3D printing facility, stimuli, stimuli-responsive materials, interaction mechanism, and mathematical modelling [23]. The term 4D printing was introduced for 3D-printed stimuli-responsive objects that undergo shape changes over time, with time considered as the fourth dimension [24]. When 3D printing is not involved in fabricating shapeshifting objects, time is still involved, as these objects require time to respond to stimuli and ultimately achieve their shape transformation. When it comes to understanding the materials’ behaviours, characterising them is essential for designing effective shapeshifting mechanisms. For example, in shape memory polymers, the softening temperature of the polymer transition segment is one of the most critical information [25]. By contrast, in smart hydrogels, the key information is often the swelling ratio and the critical solution temperature [26]. Any stimuli-responsive shapeshifting materials must meet specific criteria to enable the shapeshifting process.

Hydrogels are widely used in biomedical applications due to their outstanding properties of biodegradability, biocompatibility, hydrophilicity, superabsorbancy, viscoelasticity, softness, and fluffiness [27]. When comparing synthetic hydrogels and natural hydrogels, natural hydrogels, including alginate, gelatine, hyaluronic acid, and chitosan, have high biocompatibility, high biodegradability, and often lower mechanical strength, which limits their use in load-bearing applications [28]. On the other hand, synthetic hydrogels can be customised more readily for their biocompatibility, degradation rate, and mechanical properties [29]. Poly (N-vinylcaprolactam) (PNVCL) and poly (N-isopropylacrylamide) (PNIPAm) are two extremely popular temperature-responsive synthetic hydrogels that exhibit a lower critical solution temperature (LCST) within the physiological range [30,31]. A stronger hydrogel can enhance the stability of the shapeshifted structure; hence, following our previous study, where a mechanically improved temperature-responsive hydrogel was developed [32], we are going to demonstrate the outcome of an investigation on the shapeshifting behaviours exhibited by the developed hydrogel. The incorporation of NIPAm has been proven to improve the structural integrity of NVCL hydrogels in multiple cycles of pulsatile swelling without significantly altering the hydrogel’s LCST, which is close to the physiological range. In fact, temperature-responsive hydrogels offer advantages such as simplicity and ubiquity of temperature as a trigger, biological relevance, reversible and tunable response, and minimal invasiveness in biomedical applications, when compared to other stimulus modalities. The ability of temperature-responsive hydrogels to respond to both water and temperature makes them highly suitable for development as smart biomaterials [33]. The development of smart biomaterials enables hydrogels to achieve potential multifunctionality driven by their underlying chemistry [34]. In our investigation, bilayers made of a hydrogel layer with multiple thicknesses of flexible polymer layer were explored. The swelling of the hydrogel was assessed at multiple temperatures above and below the hydrogel’s LCST. We discovered that the hydrogel bilayers showed an exponential shapeshifting response within the tested temperature range. Additionally, we also validated the shapeshifting response using a gripper model, highlighting its promising potential as a smart bioactuator. 

Many modern surgical instruments are operated by cables, which are associated with high costs [35]. In addition to being cost-effective, the softness of hydrogels helps prevent damage to tissues, muscles, and blood vessels, especially when compared to rigid materials such as stainless steel and titanium [36]. A temperature-responsive hydrogel gripper that changes shape could be inserted through a small incision in a compact form. Once it reaches body temperature, it expands and releases the tissue during the transplantation process. Its potential to reduce tissue damage makes it suitable for the handling, positioning, and securing of delicate tissues during the transplantation process, hence improving surgical outcomes. While the use of shapeshifting hydrogels is still relatively new, the findings of this work not only showcase concepts but also contribute to the future advancement of temperature-responsive shapeshifting hydrogels in potential minimally invasive surgery applications.

## 2. Materials and Methods

### 2.1. Materials Used in This Study

The hydrogel formulation consists of the following materials: N-vinylcaprolactam (NVCL) monomer (M_w_ = 139.19 g/mol) with a storage temperature between 2 and 8 °C was purchased from Sigma-Aldrich Ireland (Dublin, Ireland). Another monomer, N-isopropylacrylamide (NIPAm) (M_w_ = 113.16 g/mol) with a storage temperature of 0 and 10 °C, was acquired from TCI Europe (Paris, France). The chemical crosslinker, poly (ethylene glycol) dimethacrylate (PEGDMA) (M_w_ = 550 g/mol) was obtained from Sigma-Aldrich Ireland (Dublin, Ireland). The photoinitiator 4-(2-hydroxyethoxy) phenyl-(2-hydroxy-2-propyl) ketone (Igracure^®^ 2959 Ciba Corp., New York, NY, USA) was bought from Ciba Specialty Chemicals (Basel, Switzerland). Bentonite nanoclay (NCB) (particle size ≤ 25 µm) as an additive was obtained from Sigma-Aldrich Ireland (Dublin, Ireland). The commercial photocurable resin, Elastic 50A V1 resin (Formlabs Company, Boston, MA, USA), was purchased from Formlabs GmbH (Berlin, Germany).

### 2.2. Silicone Mould Preparation

The mould inserts were 3D-modelled using the CAD software, CREO Parametric 10.0.0.0 (PTC Inc., Boston, MA, USA). The exported .STL files were further handled using the Cura 5.6.0 software for the 3D printing performed subsequently using an FDM printer, Ultimaker 2+ (Ultimaker, Utrecht, The Netherlands). RS Pro polylactic acid (PLA) filament (RS Group plc, London, UK) was attained from RS Radionics (Dublin, Ireland). The dimensions of the mould inserts prepared are listed in Table 1. 

The silicone mixing materials, Silastic 3481 base and 34 series fast catalyst were acquired from W. P. Notcutt Limited (Surrey, UK). Briefly, 90 wt% of Silastic 3481 base and 10 wt% of 34 series fast catalyst were mixed until homogeneous. The mould inserts were glued to the flat surface, and the silicone mixture was poured over them and left to cure overnight.

### 2.3. Synthesis of Hydrogels

Free-radical polymerisation was performed using a UV-irradiation chamber (Dr. Gröbel UV-Elektronik GmbH, Ettlingen, Germany) that contained 20 UV tubes and provided a spectral range of UVA between 315 and 400 nm at an average intensity of 10–13.5 mW/cm^2^. The bottle that contained frozen NVCL was immersed in a water bath of 50 °C until the content turned into liquid. Using a magnetic stirrer at 150 rpm and 50 °C, the materials were pre-mixed until homogeneous following the compositions listed in Table 2. The mixture was subsequently pipetted into impression-containing silicone moulds. The circular samples were UV-cured for 20 min (10 min on each face) and then oven-dried at 50 °C for 24 h to remove any unwanted moisture and residual monomer.

In the fabrication of bilayer structures, the UV-irradiation chamber was operated for 10 min on each addition of material, i.e., the NVCL mixture was cured first, and then the non-swelling Elastic 50A V1 resin was added onto the cured xerogel for another 10 min of UV irradiation. Listed in Table 3 and Table 4 are the bilayer samples produced with different volume combinations of both materials.

### 2.4. Experiment Procedures

The experiment began with conducting the swelling of circular discs at multiple temperatures. A Salvis LAB vacuum oven (Rotkreuz, Switzerland) was utilised to create the temperature environment necessary for the swelling process. In triplicate, at each temperature, a circular disc was immersed in distilled water within an individual Petri dish (Thermo Fisher Scientific, Waltham, MA, USA) with a diameter of 90 mm and a height of 15.9 mm. Prior to every measurement, the water temperature was measured using a thermometer. After 72 h of swelling, the circular discs were removed from the water and patted dry to remove surface water, and the density and weight were measured using a Rolbatch RBDT-01 density balance (Rolbatch GmbH, Brandenburg, Germany). The swelling ratio of the samples was calculated using Equation (1), where *W_t_* represents the weight of the swollen gels at a predetermined time, and *W_d_* represents the dry weight of the gels. The collected data were analysed, and the swelling ratio and density were plotted against temperature on a graph.

(1) $$SwellingRatio\left(\%\right)=\frac{W_{t}-W_{d}}{W_{d}}\times 100$$

**Figure 1:**
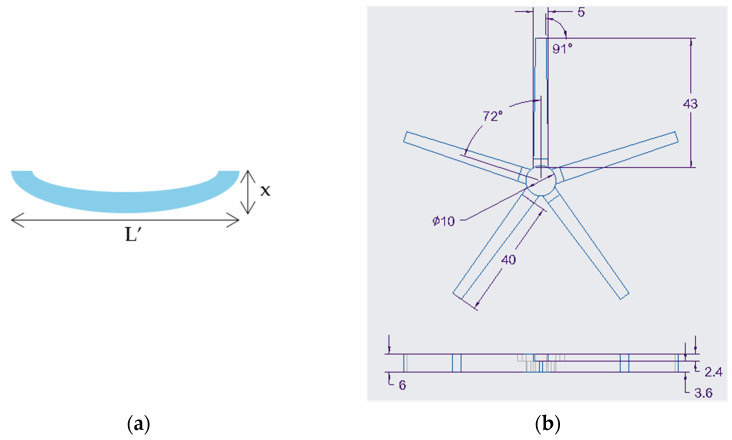
(**a**) Schematic of the dimensions measured for bilayer samples; (**b**) top and side views of the gripper and its dimensions in mm (active layer with a thickness of 2.4 mm and passive layer with a thickness of 3.6 mm).

The swelling of the bilayer samples following Table 3 was thereafter performed at multiple temperatures. It was discovered that sample 40C had the desired response; hence, additional swelling experiments were performed on samples with an identical ratio of active layer to passive layer, as indicated in Table 4. Due to curvature, the change in horizontal length (*L’*) and the change in the vertical displacement (*x*) of the bilayer samples were measured, as illustrated in Figure 1a. The measured data were used in calculating the radius of the circular arc due to the shapeshifting of the samples, using Equation (2). For samples that curled into a complete circle, Equation (3) was used to calculate the radius, where *d* represents the diameter of the circle. The results were analysed to identify any patterns in their responses.

(2) $$Radius\;\left(mm\right),\;\;r=\frac{{L^{\prime }}^{2}}{8x}+\frac{x}{2}$$

(3) $$Radius\;\left(mm\right),\;\;r=\frac{d}{2}$$

### 2.5. Shapeshifting of a Gripper Model

The gripper mould insert was 3D-modelled and 3D-printed using the same software and printer. The dimensions of the gripper mould insert are shown in Figure 1b. A silicone mould was then prepared using the mould insert. Under the same active-layer-to-passive-layer ratio of 1:1.5, the NVCL mixture was UV-cured primarily for 10 min, and subsequently, the Elastic 50A V1 resin was added onto the cured xerogel to UV-cure for another 10 min. The prepared gripper was secured to a wire by piercing and creating a knot through its centre and then submerged in distilled water at room temperature with a 20 mL volumetric flask placed underneath for 72 h to ensure swelling to equilibrium. Following that, the water temperature was kept at 30 °C for 72 h. The weight of the volumetric flask containing distilled water was measured at 42.5 g, and the body’s widest diameter was measured at 36.5 mm.

## 3. Results and Discussion

### 3.1. Photopolymerisation

**Figure 2:**
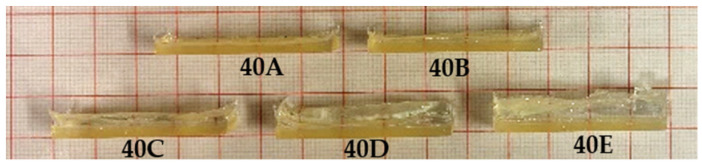
Photopolymerised bilayer samples with different active-layer-to-passive-layer ratios: 40A (1:0.5), 40B (1:1), 40C (1:1.5), 40D (1:2), and 40E (1:2.5).

All circular and rectangular samples were fabricated successfully through photopolymerisation. During the photopolymerisation process, the photoinitiator Irgacure 2959 absorbed UV light and formed free radicals, which initiated the polymerisation of the resin. While our previous study demonstrated the 3D printing of NVCL resin [37,38], utilising the photopolymerisation technique offers the advantage of maximising output in a significantly shorter period of time. The fabricated circular samples were similar to those in the previous study [32], and the fabricated rectangular bilayer samples are shown in Figure 2. It can be noticed that the bottom active layer made of the NVCL resin was cured in a well-defined, regular shape. However, the upper passive layer made of the Elastic 50A V1 resin did not cure into a regular shape, which was likely due to the highly viscous properties of the resin that caused stronger cohesion to the walls of the mould. Furthermore, the warping of the passive layer can be noticed with the increasing volume of the Elastic 50A V1 resin. This occurred because the UV irradiation in the chamber was applied from the top, causing the resin to cure from the top down and ultimately leading to noticeable warping in thicker samples. The active layer bonded with the passive layer through physical interaction, following our previously proven method, without the application of any adhesive agent [26]. All fabricated samples were dried in the oven before proceeding with the swelling tests.

### 3.2. Characterisation of Hydrogel for Shapeshifting

Before programming any shapeshifting material, it is essential to first characterise its shapeshifting behaviour [39]. PNVCL as one of the negatively temperature-responsive hydrogels, has important characteristics, including LCST, phase transition behaviour, thermal responsiveness, biocompatibility, and mechanical properties, that allow them to be applied in areas such as drug delivery, tissue scaffolding, and actuators [40]. Although hydrogels typically exhibit poor mechanical properties [41], the NVCL resin formulation employed in this study was derived from preceding research that optimised the hydrogel mechanical strength [32,42]. A more robust gel facilitates easier performance of the shapeshifting function, like having a stronger muscle. Though we characterised the formulation employed in this study previously, the density and swelling ratio of the hydrogels at various temperatures were not investigated, especially at temperatures around the hydrogel LCST. Hence, the density and swelling ratio of the circular samples were measured at 18, 28, 29, 30, 31, 32, 33, 35, and 49 °C. The temperatures at which the density and swelling ratio were measured were the actual water temperature instead of the oven-set temperature. The reason was to avoid recording inaccurate temperatures due to the environment, and the measurements were performed using a thermometer. 

**Figure 3:**
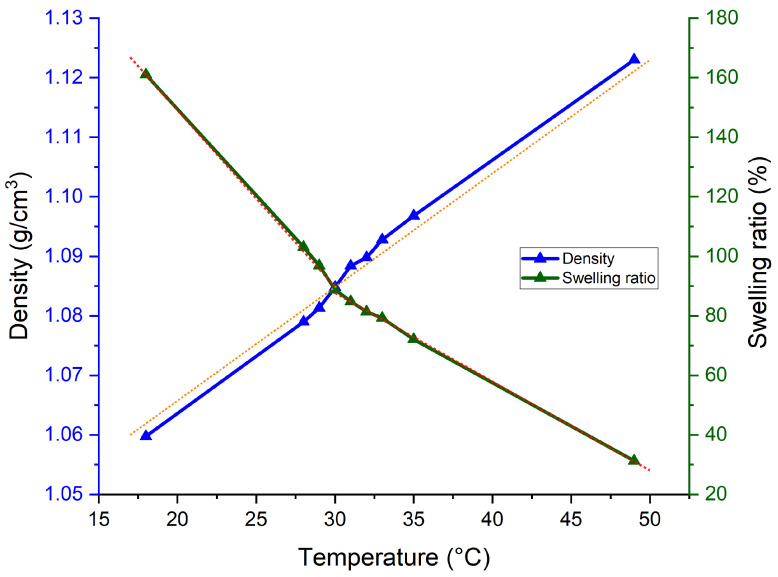
Density and swelling ratio of the circular hydrogel samples at various temperatures. The dotted lines represent the best-fit lines for the data.

**Figure A1:**
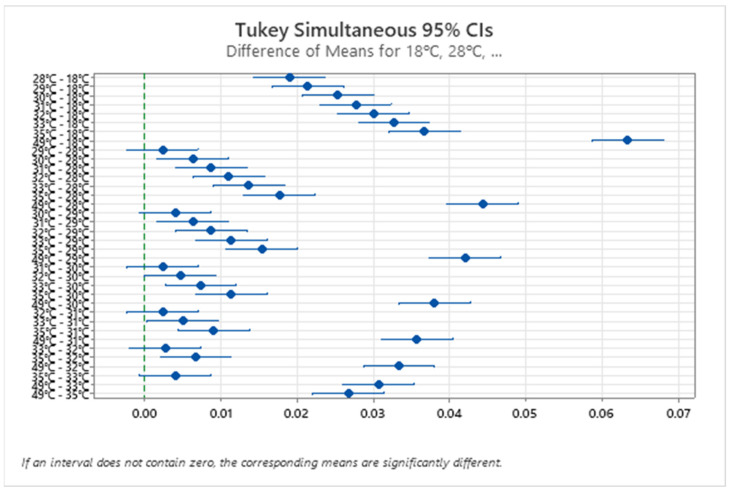
Tukey simultaneous comparison graph of the collected density data.

Figure 3 shows the change in the density and swelling ratio of the hydrogel due to different temperatures. The dotted straight lines represent the lines of best fit to two different sets of data. It can be observed from the graph that, across the x-axis, the resulting density of the hydrogel exhibited a linear relationship, indicating a direct proportionality between density and temperature. On the other hand, the swelling ratio curve exposed two different gradients before and after the temperature around the LCST. The swelling ratio decreased more significantly from 18 to 30 °C compared to the decrease observed from 31 to 49 °C. Generally, when PNVCL is in an aqueous form above the LCST, the polymer changes its hydrophilic to hydrophobic balance and expels water molecules, which causes phase transition to occur [43]. Hence, the findings indicate that in our chemically crosslinked hydrogels, which do not dissolve in water due to the existence of covalent bonds, the swelling ratio decreased with an increase in temperature from 18 to 49 °C. Moreover, the swelling phenomenon of NVCL hydrogels following two different rates above and below their LCST could be due to the change in the hydrogel temperature sensitivity [44]. Below the LCST, due to stronger hydrophilic interactions, the rate of change in swelling was more distinct, whilst above the LCST, the stronger hydrophobic interactions led to a slower rate of change with temperature caused by a collapsed polymer network. Overall, at lower temperatures, hydrogel polymer chains were in an extended conformation, allowing for high water absorption, whereas at higher temperatures, the polymer chains shrank, decreasing water absorption. As the volume of hydrogels decreased, their density increased with rising temperatures. Table A1 contains the density data of the circular discs measured at various temperatures. With an overall difference in measured data of 0.08 g/cm^3^, a one-way ANOVA was performed. It was found that the difference between the collected density data was statistically significant (*p*-value < 0.05), as shown in Table A2. Subsequently, Tukey’s honestly significant difference (HSD) test was carried out to determine which specific pairs of temperatures have significantly different means. In Figure A1, all pairs of temperatures show significantly different means, except the pairs with 1 °C difference. This suggests that the density of the gels shows an influential difference when the temperature difference is sufficiently large.

### 3.3. Optimising Bilayer Sample Thickness Ratio

To design an effective actuator, a more significant change in conformation would be the preferable feature. In this section, the optimised ratio between the passive and active layers is explored. The passive layer made of the Elastic 50A V1 resin in different volumes was UV-cured on top of the hydrogel active layer of constant volume. Following the study of the circular hydrogel samples, it was established that the rate of change in the swelling ratio was more significant below the LCST. Therefore, bilayer samples of different layer thickness ratios were swollen at 20 and 30 °C, to find out the best volume of the passive layer combined with 0.6 mL of the hydrogel active layer. 

**Figure 4:**
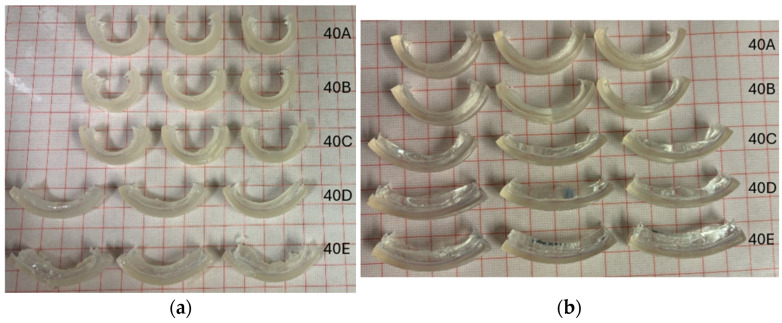
Swollen bilayer samples at (**a**) 20 °C and (**b**) 30 °C. The passive layer volume started at 0.3 mL (top) to 1.5 mL (bottom).

Shown in Figure 4 are the photos taken of the bilayer samples swollen to equilibrium at 20 and 30 °C. It can be observed that the bilayer samples showed more significant bending when swelling at 20 °C, whereas at 30 °C, the bending effect became less significant. This was due to the decrease in the hydrophilicity of the hydrogel polymer network at 30 °C. Furthermore, it can also be noticed that with an increase in the thickness of the passive layer, the bending effect was diminished. When keeping the active layer volume constant, the amount of force generated for bending due to the swelling of hydrogels remained the same. Hence, the bending degree solely depended on the passive layer volume, and the diminishing bending effect was due to the increase in the passive layer stiffness as the layer thickened. 

**Figure 5:**
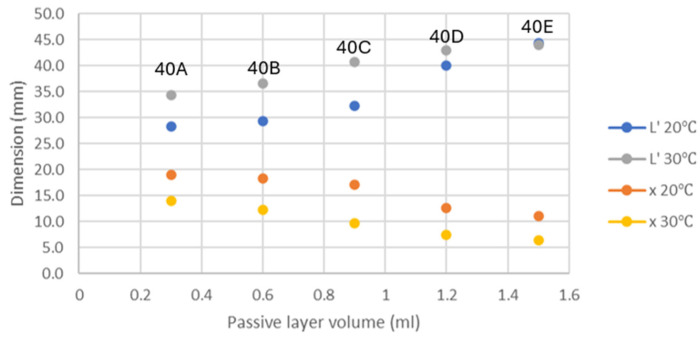
The dimensional change in the fully swollen bilayer samples at 20 and 30 °C.

All bilayer samples were measured for the dimensional change, and the sets of data are plotted in Figure 5. From the graph, the comparison of the change in horizontal length (L’) and vertical displacement (x) indicated that the thickness of the passive layer influenced the dimensional change. With 1.5 mL of the passive layer, the bilayer samples showed the least significant difference in L’ and x at both temperatures. The difference in the dimensional change became more significant with the decreasing volume of the passive layer. However, the most significant difference in dimensional change was found in the bilayer structures containing 0.9 mL of the passive layer. When compared to bilayer samples made of 0.3 and 0.6 mL of the passive layer, 0.9 mL of the passive layer appeared to provide the optimal response to the bending of the structure. This could be because, at 0.9 mL of the passive layer, the passive material provided more force than hydrogel to pull back the curvature at lower hydrogel swelling, while at 20 °C, the force generated by the hydrogel was still great enough to overcome the stiffness of the passive material, resulting in very significant bending. As a result, the ratio of 1:1.5 (active layer to passive layer) was selected to continue the rest of the study. 

### 3.4. Shapeshifting Response of Bilayer Sample with Different Dimensions

The shapeshifting response of bilayer samples made of a 1:1.5 active-layer-to-passive-layer ratio was subsequently investigated. Using the same ratio, bilayer samples of different dimensions were tested, i.e., samples 40C, 60C, 80C, and 100C. Samples 40C, 60C, and 80C had the same width, whereas sample 100C had a narrower width of 2 mm instead of 8 mm. This variation was intended to explore whether width influences the shapeshifting response of the bilayer structure. At 20, 25, 30, 40, and 50 °C, the bilayer samples of different dimensions were immersed in distilled water for the investigation.

Initially, we assumed that the shapeshifting response of the bilayer samples would follow a similar response as the bimetallic strips. In general, bimetallic strips are made of two different metals bonded together, which have different coefficients of thermal expansion. This feature allows the strip to bend when exposed to temperature-induced thermal stresses due to different thermal expansions [45]. In our case, the thermal expansion could be replaced by the expansion due to swelling, and the bilayer structure would exhibit two different coefficients of swelling expansion below and above the LCST. However, from the results obtained, the bending of the bilayer samples due to swelling did not follow a linear response.

**Figure 6:**
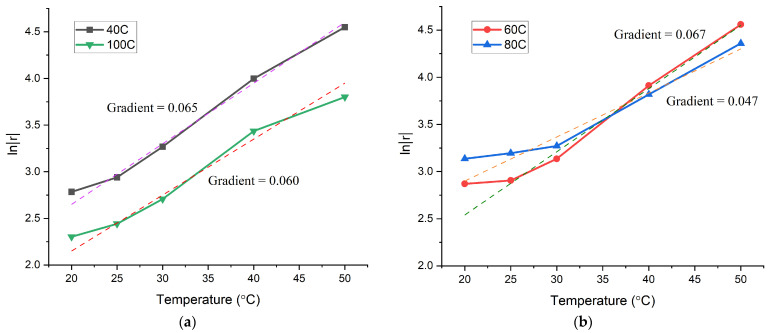
Natural logarithm of radius against swelling temperature: (**a**) samples 40C and 100C; (**b**) samples 60C and 80C. The dotted lines represent the best-fit lines for the data.

The radius (r) was calculated using Equation (2), and Equation (3) was used for the bilayer samples that curled into a complete circle. The obtained sets of data against swelling temperature were plotted in graphs, which are shown in Figure 6. It was discovered that the bilayer samples shapeshifted exponentially due to the change in temperature. The curves representing samples 40C, 60C, 80C, and 100C generally exhibited a straight-line behaviour on the exponential graphs. From each of the curves, at 20 °C, the natural logarithm of radius deviated most from the lines of best fit, which could be due to the stiffness of the passive material that restricted the bending to some degree. Although we concluded in the previous section on optimising the bilayer sample thickness ratio that 0.9 mL of the passive layer worked best, there was inevitably the stiffness of the passive layer that continuously counteracted the force produced by the hydrogel. Furthermore, the gradients calculated for the lines of best fit of the curves for samples 40C, 60C, and 100C were within a similar range. The curve for sample 80C, however, resulted in a deviated gradient. This could be due to the geometry as a factor, which affected the bending response. When compared to sample 40C, sample 100C had a width of 2 mm instead of 8 mm, and this greatly reduced any effect of width on the bending response; hence, in Figure 6a, both curves’ line of best fit is almost parallel to one another. In sample 80C, the length was twice that of sample 40C while maintaining the same width, and that resulted in an evident, reduced rate of change in the curvature radius. 

**Figure 7:**
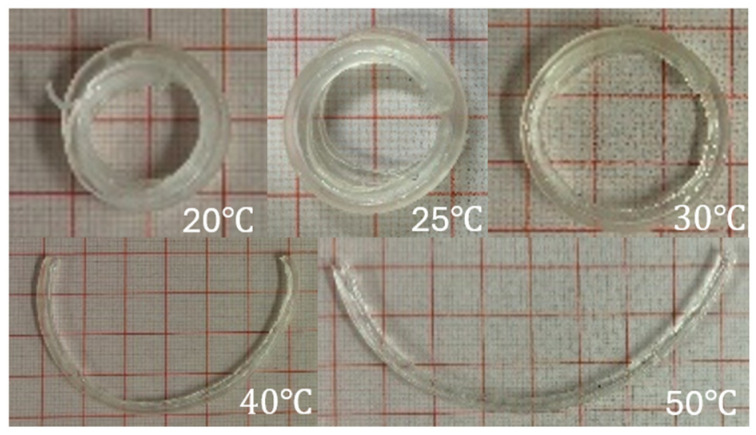
Swollen 100C sample at various temperatures.

Overall, according to the calculated gradients, the rate of change in radius decreased in the following order by temperature: samples 100C, 40C, 60C, and 80C. The possible reason for this could be the increased stiffness due to an increased sample size. The shapeshifting of sample 100C into a circle at lower temperatures is shown in Figure 7. Usually, a material becomes easier to bend when elongated. In a bilayer structure, both the passive and active layers had the same length. However, the passive layer exerted more force to resist bending than the hydrogel force exerted to shift the shape. We can therefore conclude that the bilayer structure geometry also played a part in affecting their shapeshifting behaviour. As a result, the findings from this study proved that the bending effect of our bilayer samples can be explained using Equation (4), where *α* represents the coefficient of swelling expansion, *G* represents the geometry coefficient, and *T* represents the temperature.

(4) $$Radius,\;r=e^{\alpha GT}$$

### 3.5. Shapeshifting Gripper

**Figure 8:**
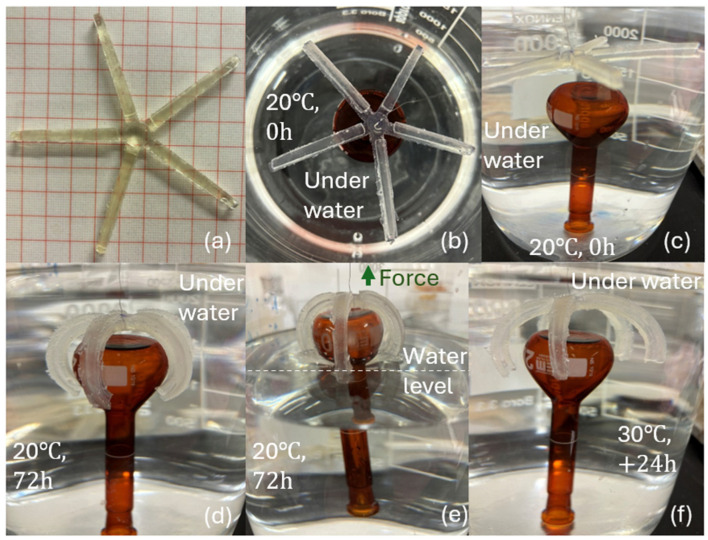
Fabricated bilayer gripper model: (**a**) before swelling; (**b**) immersed in distilled water at 0 h (top view); (**c**) swelling at 0 h (side view); (**d**) swelling to equilibrium at 20 °C (72 h); (**e**) pulling object out from the water (from (**d**)); (**f**) swelling to equilibrium at 30 °C (further 24 h).

To verify the shapeshifting ability of the PNVCL bilayer structure as an actuator, a gripper model was produced. Under the 1:1.5 active-layer-to-passive-layer ratio, the fabricated gripper was weighed at 5.4 g in its non-swollen state. Shown in Figure 8 are the photos taken of the gripper fabricated and swollen at 20 and 30 °C. Although hydrogels typically weaken with increasing swelling, the flexible material acting as a passive layer could provide the necessary support to the entire structure for the shapeshifting to occur. From the result, the gripper weighing 5.4 g can hold and lift the volumetric flask weighing 42.5 g from the water. Although there was buoyancy involved, the gripper proved to be remarkably strong, capable of carrying a weight several times greater than its own. In addition, at 30 °C, when the hydrogel swelling ratio was lower, the gripper opened, and the volumetric flask was released. Overall, NVCL hydrogel combined with another material in a bilayer structure shows promise as a smart bioactuator due to its superior benefits.

## 4. Conclusions

Through UV irradiation, the bilayer samples comprising an active hydrogel layer and a passive flexible layer were successfully synthesised. The Elastic 50A V1 resin was found to be dimensionally stable at low volume through the UV technique. The interactions between the active and passive layers were adequate in performing simple shapeshifting mechanisms; however, further bonding techniques may need to be explored for multiple cycles of shapeshifting back and forth to avoid layer delamination. From the hydrogel density and swelling characterisation across a range of temperatures, it was found that the density exhibited a direct proportionality with the temperature, whereas the rate of change in the swelling ratio with temperature was found to be different before and after the hydrogel LCST, due to the change from dominant hydrophilic interactions to stronger hydrophobic interactions. Furthermore, through screening, a passive layer volume of 1.5 times that of the active layer was found to exhibit the most pronounced shapeshifting characteristics. A higher volume of the passive layer can restrict the shapeshifting effect due to higher stiffness and vice versa. Nonetheless, if the passive layer volume is too low, it would not generate enough force to pull back the structure under the low swelling of hydrogel. By fabricating bilayer samples of different dimensions, it was discovered that the structure geometry affected the shapeshifting behaviour due to the change in stiffness. The overall bending of the bilayer samples was found to be following an exponential response. Lastly, a temperature-responsive gripper made of a bilayer structure was fabricated to verify the shapeshifting ability. The gripper successfully picked up an object that was nearly 8 times more than its weight and released the object at an elevated temperature. To conclude, the findings from this study contribute to the development of temperature-responsive hydrogel actuators and highlight the essential features of smart materials crucial for designing effective actuators. Future work including a deeper investigation into the geometric features that impact the bending response will need to be carried out to improve our understanding of the bilayer structure.

## Figures and Tables

**Table 1:** Dimensions of the mould inserts used in this study.

Mould Inserts	Dimensions (mm)
Circular	30 × 4 (Φ × H)
Rectangular 40	40 × 8 × 10 (L × W × H)
Rectangular 60	60 × 8 × 10 (L × W × H)
Rectangular 80	80 × 8 × 10 (L × W × H)
Rectangular 100	100 × 2 × 3 (L × W × H)

**Table 2:** Compositions of the materials.

NVCL (wt%)	NIPAm (wt%)	PEGDMA (wt%)	Irgacure 2959 (wt%)	NCB (ratio wt%)
59.94	29.97	9.99	0.10	3.00

**Table 3:** Material compositions of the bilayer samples with 40 mm length.

Sample	Sample Dimensions (L × W) (mm)	Ratio (Active Layer to Passive Layer)	Volume (mL)	
			NVCL Mixture	Elastic 50A V1
40A	40 × 8	1:0.5	0.6	0.3
40B		1:1	0.6	0.6
40C		1:1.5	0.6	0.9
40D		1:2	0.6	1.2
40E		1:2.5	0.6	1.5

**Table 4:** Material compositions of the bilayer samples with 60, 80, and 100 mm lengths.

Sample	Sample Dimensions (L × W) (mm)	Ratio (Active Layer to Passive Layer)	Volume (mL)	
			NVCL Mixture	Elastic 50A V1
60C	60 × 8	1:1.5	0.9	1.35
80C	80 × 8		1.20	1.80
100C	100 × 2		0.24	0.36

## Data Availability

The original contributions presented in the study are included in the article, further inquiries can be directed to the corresponding authors.

## Appendix A

**Table A1:** The density of the circular discs measured at various temperatures.

		Temperature (°C)								
		18	28	29	30	31	32	33	35	49
Density (g/cm^3^)	1	1.060	1.079	1.084	1.084	1.087	1.090	1.092	1.096	1.125
	2	1.062	1.080	1.078	1.086	1.088	1.091	1.092	1.096	1.121
	3	1.058	1.078	1.082	1.086	1.088	1.089	1.094	1.098	1.124

**Table A2:** Analysis of variance (ANOVA) of the collected density data.

Source	DF	Adj SS	Adj MS	F-Value	*p*-Value
Factor	8	0.006793	0.000849	318.41	0.000
Error	18	0.000048	0.000003		
Total	26	0.006841			
